# The Effect of Conjugated Linoleic Acid Supplementation on Densitometric Parameters in Overweight and Obese Women—A Randomised Controlled Trial

**DOI:** 10.3390/medicina59091690

**Published:** 2023-09-21

**Authors:** Małgorzata Jamka, Agata Czochralska-Duszyńska, Edyta Mądry, Aleksandra Lisowska, Katarzyna Jończyk-Potoczna, Judyta Cielecka-Piontek, Paweł Bogdański, Jarosław Walkowiak

**Affiliations:** 1 Department of Pediatric Gastroenterology and Metabolic Diseases, Poznan University of Medical Sciences, Szpitalna Str. 27/33, 60-572 Poznań, Poland; mjamka@ump.edu.pl; 2 Department of Physiology, Poznan University of Medical Sciences, Święcickiego Str. 6, 61-781 Poznań, Poland; aczochralska-duszynska@ump.edu.pl (A.C.-D.); emadry@ump.edu.pl (E.M.); 3 Department of Pediatric Diabetes, Auxology and Obesity, Poznan University of Medical Sciences, Szpitalna Str. 27/33, 60-572 Poznań, Poland; alisowska@ump.edu.pl; 4 Department of Pediatric Radiology, Poznan University of Medical Sciences, Szpitalna Str. 27/33, 60-572 Poznań, Poland; jonczyk@ump.edu.pl; 5 Department of Pharmacognosy and Biomaterials, Poznan University of Medical Sciences, Rokietnicka Str. 3, 60-806 Poznań, Poland; jpiontek@ump.edu.pl; 6 Department of Treatment of Obesity, Metabolic Disorders and Clinical Dietetics, Poznan University of Medical Sciences, Szamarzewskiego Str. 84, 60-569 Poznań, Poland; pbogdanski@ump.edu.pl

**Keywords:** bone mineral content, bone mineral density, densitometry, obesity

## Abstract

*Background and Objectives*: Conjugated linoleic acid (CLA) can improve bone health in animals, yet the effects on humans have not been consistent. Therefore, this parallel randomised controlled trial aimed to assess the effect of CLA supplementation on bone mineral density (BMD) and content (BMC) in overweight or obese women. *Materials and Methods*: The study population included 74 women who were divided into the CLA (*n* = 37) and control (*n* = 37) groups. The CLA group received six capsules per day containing approximately 3 g of cis-9, trans-11 and trans-10, cis-12 CLA isomers in a 50:50 ratio. The control group received the same number of placebo capsules that contained sunflower oil. BMC and BMD at total body, lumbar spine (L1–L4), and femoral neck were measured before and after a three-month intervention. *Results*: The comparison of BMC and BMD for the total body, lumbar spine (L1–L4), and femoral neck before and after the intervention showed no differences between the groups. However, a within-group analysis demonstrated a significant increase in BMC (*p* = 0.0100) and BMD (*p* = 0.0397) at lumbar spine (L1–L4) in the CLA group. Nevertheless, there were no significant differences between the CLA and placebo groups in changes in all analysed densitometric parameters. *Conclusions*: Altogether, three-month CLA supplementation in overweight and obese women did not improve bone health, although the short intervention period could have limited our findings, long-term intervention studies are needed. The study protocol was registered in the German Clinical Trials Register database (ID: DRKS00010462, date of registration: 4 May 2016).

## 1. Introduction

Many naturally occurring food substances possess functional properties [1], such as conjugated linoleic acid (CLA). CLA is a set of various fatty acids, including geometric and positional isomers of linoleic acid. While sharing the same carbon chain length as linoleic acid, CLA differs in double bond arrangement [2]. Instead of methylene-separated carbon atoms, CLA features conjugated double bonds, separated by a one carbon–carbon bond instead of multiple bonds. These conjugated bonds can exist in both cis and trans configurations. Several different isomers of CLA have been identified, including 11-trans and 9-cis or 10-trans and 12-cis [3]. Among them, the 11-trans and 9-cis isomer is the most common in the human diet [2,3]. Anaerobic microorganisms primarily make CLA from ruminants, as CLA is most commonly present in milk products and meat, while eggs and poultry contain less CLA [4].

Positive effects of CLA on human health have been reported [5]. CLA has shown potent anticarcinogenic [6] and antiatherogenic effects [7], may modulate immune activity [8], and improve body composition and anthropometric parameters in overweight or obese subjects [9]. Additionally, it is suggested that the anti-inflammatory effect of CLA may affect bone mass [10,11]. CLA decreases prostaglandin E2 production [12,13,14] and modulates calcium absorption, which also may affect bone health [15]. Indeed, animal studies have observed improvements in bone health after CLA supplementation [16,17]. However, the effects of CLA on bone mass in humans have provided both positive impact of CLA intake on bone mineral density (BMD) [18], but others reported no effect of CLA supplementation on bone health [19,20,21,22] or potential adverse effects on bone mineral content (BMC) [23].

Therefore, the study aimed to assess the effect of three-month CLA supplementation on BMD and BMC in overweight or obese women.

## 2. Materials and Methods

### 2.1. Study Design

As described previously [9,24,25,26,27], the study was designed as a parallel, double-blind, randomised controlled trial, registered with the German Clinical Trials Register database (ID: DRKS00010462, date of registration: 4 May 2016) [28]. The study protocol was approved by the Poznan University of Medical Sciences Ethics Committee (protocol code: 606/12, date of approval: 14 June 2012; protocol code: 453/13, date of approval: 9 May 2013; protocol code: 358/14, date of approval: 3 April 2014; protocol code: 398/15, date of approval: 9 April 2015). Before enrolling in the study, we obtained written informed consent from all participants. The project was performed according to the Declaration of Helsinki [29]. The manuscript was written according to the consolidated standards of reporting trials (CONSORT, see Table S1, Supplementary Materials) [30].

### 2.2. Inclusion and Exclusion Criteria

The screening of the potential participants was performed at the Department of Internal Medicine, Metabolic Disorders and Hypertension, Poznan University of Medical Sciences, Poland. The recruitment was carried out from July 2014 to May 2015. The inclusion and exclusion criteria are listed in Table 1.

### 2.3. Intervention Protocol

The participants were divided into the CLA group and the placebo group. During the intervention period (three months), both groups received six capsules per day, each containing 0.5 g of substrate. Capsules in the CLA group contained 80% CLA (50% of cis-9, trans-11 and 50% of trans-10, cis-12 isomers), while capsules for the placebo group contained sunflower oil. Capsules for the CLA and placebo groups looked identical and were manufactured by the Olimp Laboratories company (Pustynia, Poland). The oils did not differ in energy value. A comparison of fatty acids composition between CLA and sunflower oils is presented in Table 2. The subjects were instructed not to modify their dietary habits or physical activity during the intervention period.

### 2.4. Outcomes

The primary outcome of the study was the effect of CLA supplementation on the digestion of starch and lipids [27]. Here, we reported the effects of CLA intervention on secondary outcomes, which included the impact on densitometric parameters.

### 2.5. Anthropometric Parameters

The following anthropometric parameters were assessed: body weight and height, waist and hip circumference, and BMI. All parameters were measured in the fasting state, in underwear and barefoot. The body weight and height were assessed using a scale with a stadiometer (Radwag, Random, Poland). The BMI was calculated [31]. The hip and waist circumferences were assessed using a Seca scale (Hamburg, Germany).

### 2.6. Densitometric Parameters

All subjects included in the study had their densitometry parameters assessed before and after the intervention using Hologic Discovery Wi (Bedford, MA, USA) equipment. The BMC and BMD at total body, lumbar spine, and femoral neck and fat mass were evaluated. The assessment was performed at the Department of Pediatric Gastroenterology and Metabolic Diseases, Poznan University of Medical Sciences. During the measurements, the subjects were asked to dress in lightweight clothing and to remove their shoes and any metal objects. Before the measurement, subjects avoided physical activity. The measurement was performed in the supine position and was of approximately 15 min duration. The radiation dose ionizing agent ranged from 0.5 mGy to 0.7 mGy. The intra- and inter-individual coefficients of variation for bone mass were less than 1%.

### 2.7. Randomisation and Blinding

Block randomisation was performed. Participants were assigned to CLA and placebo groups based on a computer-generated randomisation list (allocation ratio: 1:1). The study participants, outcome assessors, and statistician were blind to the allocation of treatment.

### 2.8. Minimum Sample Size

The sample size was determined using the Statistica 12 PL software (TIBCO Software Inc., Palo Alto, CA, USA). By considering the primary outcome, it was calculated that 74 subjects would need to be enrolled in the study to achieve an 80% power level, with α and β values of 0.05 and 0.2, respectively.

### 2.9. Statistical Analysis

The statistical analyses were conducted using the Statistica 12 PL software (TIBCO Software Inc., Palo Alto, USA). For each parameter, the mean, standard deviation (SD), median, and interquartile range (IQR) were calculated. The Shapiro–Wilk test was used to determine data compliance with the normal distribution. We considered a *p*-value of less than 0.05 to indicate statistical significance level. Comparisons between two unpaired groups were determined using *t*-tests (normal distribution, homogeneous variances), Cochran-Cox (normal distribution, lack of homogeneity of variance) or Mann–Whitney U-test (lack of normal distribution). In addition, a paired sample *t*-test or the Wilcoxon test were used to assess intragroup change between pre- and post-intervention values in the CLA and placebo groups.

## 3. Results

### 3.1. Participants’ Flow

**Figure 1:**
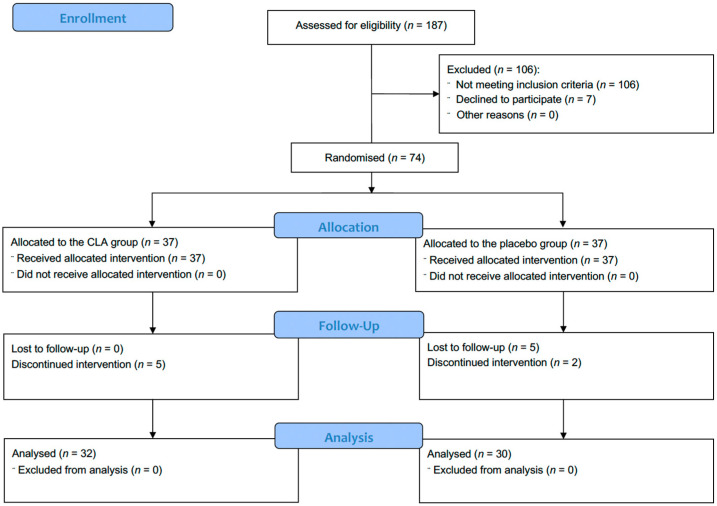
CONSORT 2010 participants flow diagram.

The participant’s flow is shown in Figure 1. A total of 187 subjects reported willingness to participate in the study. Of these, 81 women met the inclusion criteria, though seven subjects refused to participate or withdrew from the study for the following reasons: three due to lack of time, one due to abdominal pain and diarrhoea, one due to suspected ovarian tumour, one due to personal problems, and one due to difficulty cooperating. Finally, 74 Caucasian women were randomized into the CLA (*n* = 37) and placebo (*n* = 37) groups. During the intervention, 12 women withdrew from the study: five from the CLA group (three did not come to appointments, one was affected by nausea, one became pregnant) and seven from the placebo group (four missed the appointments, two were affected by nausea, one had a rash). In total, 62 participants were included in the final analysis. The anthropometric characteristics of the study population are presented in Table 3.

### 3.2. The Effect of CLA Supplementation on Densitometric Parameters

The comparison of BMC and BMD for the total body, lumbar spine (L1–L4), and femoral neck before and after the intervention showed no differences between the groups. However, a within-group analysis demonstrated a significant increase in BMC (*p* = 0.0100) and BMD (*p* = 0.0397) at lumbar spine (L1–L4) in the CLA group (Table 4). Nevertheless, the comparison of changes in analysed parameters showed no statistically significant differences between the CLA and placebo groups (Table 5).

## 4. Discussion

Here, we observed that in overweight and obese women, post-intervention BMC and BMD at lumbar spine (L1–L4) in the CLA group significantly increase compared to pre-intervention values. However, no differences in changes in densitometric parameters between groups were detected.

Excessive body weight is an important factor that may affect bone health. Previously, being overweight or obese was commonly seen as a factor contributing to higher bone density and a reduced risk of osteoporosis and fractures [32,33]. This association was attributed to both mechanical effects and the greater presence of oestrogens in adipose tissue [34]. Nonetheless, other findings indicate that an excess of fat mass may not protect humans from osteoporosis and fractures [35]. Obesity, because of its systemic inflammatory state, can disturb the bone remodelling process, leading to the dysregulation of bone homeostasis and promoting bone loss. Other factors may also play a role in this process, such as oxidative stress, insulin resistance, alterations in gut microbiota, and hormonal changes [36]. Based on these findings, we speculate that the effect of CLA supplementation on bone health might differ in subjects with excessive and normal body weight.

Previously, only a few studies evaluated the effect of CLA supplementation on densitometric parameters in overweight and obese subjects and reported no effect or negative impact on bone health [20,21,22,23]. Gaullier et al. [20] investigated the effect of CLA supplementation in a 3.4 g/d (comprising 37.5% cis-9, trans-11 and 38% trans-10, cis-12 isomers) dose for six months in overweight and obese healthy adults, and reported no effects on BMC. In another study performed by the same authors, over 12-month supplementation with triacylglycerol-bound CLA (50% cis-9, trans-11 and 50% trans-10, cis-12, 3.4 g/d of active isomers) in overweight subjects was not effective in improving bone mass, whereas CLA isomers in the form of free fatty acids (3.6 g/d of active isomers) significantly reduced the bone mineral mass. However, changes within the CLA groups were not significantly different from those within the placebo group [21]. After one year of the intervention, out of the 157 subjects who participated in the research, 134 were enrolled in an open study for the subsequent 12 months; however, there were no differences within CLA groups in bone mineral mass between pre- and postintervention values and between CLA and placebo groups [22]. Additionally, Racine et al. [23] reported that seven months of CLA supplementation (3 g/d of 80% trans-10, cis-12 and cis-9, trans-11 in equal proportion) in overweight and obese children had a negative effect on bone health, and decreased the accrual of the total body BMC.

Studies assessing the impact of CLA supplementation on bone health, which were not only focused on overweight or obese individuals, provided more ambiguous results. Some of these studies reported no effects on bone health [19,37,38,39] while in others, positive effects were observed [19,40,41]. Tarnopolsky et al. [37] examined whether adding creatine monohydrate and CLA (6 g/d, combination of 45% cis-9, trans-11 and 45% trans-10, cis-12 isomers) supplementation to resistance training affected bone mass after six months of intervention in older subjects. The authors observed that the total BMD and lumbar BMD did not change after the intervention; however, hip BMD decreased for men only after training. Similarly, Kreider et al. [19] assessed the impact of CLA supplementation (6 g/d as mixed isomers) in male athletes undertaking resistance training, and showed no statistically significant changes in bone mass. However, this study evaluated a small subject number (n = 23) over a short period (28 d), which could have limited the findings. Brown et al. [38] also observed no change in the BMD and BMC when 18 young, healthy women consumed a CLA-enriched diet (1.17 g/d) over 56 d. Furthermore, Doyle et al. [39] analysed the effect of 3 g/d (50% of cis-9, trans-11 and 50% of trans-10, cis-12 isomers) CLA supplementation in healthy adult men for eight weeks, and reported no discernible impacts on bone formation (osteocalcin and bone-specific alkaline phosphatase) or bone resorption (C-telopeptide-related fraction of type 1 collagen degradation products, N-telopeptide-related fraction of type 1 collagen degradation products, pyridinoline and deoxypyridinoline) markers. On the other hand, Brownbill et al. [18], in a cross-sectional analysis of 136 postmenopausal women, showed that the consumption of CLA was identified as s a significant predictor of Ward’s triangle BMD. Furthermore, women with CLA intake higher than median level exhibited a higher BMD of the forearm. DeGuire et al. [40] recorded that men with red blood cell cis-9, trans-11 CLA status above the median had higher whole-body BMD. This finding was confirmed in a regression analysis, which showed that red blood cell cis-9, trans-11 CLA levels influenced significantly total-body BMD. Moreover, Aryaeian et al. [41] noted that 2 g/d of 9-cis, 11-trans and 10-cis, 12-trans CLA supplementation for 12 weeks had a positive effect on telopeptides C and osteocalcin levels in subjects with rheumatoid arthritis. Furthermore, Pinkoski et al. [42] reported that 5 g/d CLA supplementation combined with resistance training decreased the bone resorption parameters (urinary cross-linked N-telopeptides of type I collagen) after 14 weeks of cross-over intervention.

Several mechanisms have been suggested to clarify the potential positive impacts of CLA on densitometric parameters. First, CLA may decrease prostaglandin E2 production through the cyclooxygenase enzyme system and, therefore, may affect bone health [12,13]. It is widely recognised that prostaglandin E2 plays an important role in regulating bone health [14]. Second, CLA may stimulate calcium absorption to be used in bone formation [15]. CLA may also affect bone health by impacting leptin expression and levels [43]. Leptin affects bone metabolism by stimulating bone marrow stromal cells and inhibiting osteoclast cells [44]. Additionally, CLA mitigates age-related bone loss by reducing inflammatory markers and expression of osteoclast cells [10,11].

In addition to nutritional status, several other factors may be responsible for the potential differences between the results of individual studies analysing the effect of CLA supplementation on bone health. The duration of the intervention, dose of CLA, age, sex, hormonal and health status may determine the effectiveness of CLA supplementation. It was estimated that the bone turnover process may last approximately six months [45]. However, no effect of CLA supplementation was observed both in studies with shorter (<6 months) [19,38,39] and longer (≥6 months) duration periods [20,21,22,37]. Moreover, no effect of CLA supplementation on bone mass was found after using low (≤3 g/d) [38,39] or high (>3 g/d) CLA doses [19,20,21,22,37]. The average intake of CLA from natural sources seems to have a malignant effect as the intake of cis-9, trans-11 isomer was approximated as 151–212 mg/d for Americans and 97.5 mg/day for British populations [46,47]. Therefore, further studies are needed to assess how age, sex, and health status may affect the findings. However, we speculate that CLA supplementation may be more effective in subjects with bone diseases [41] or postmenopausal women [39]. Isomer specificity may also determine the effectiveness of CLA supplementation on bone health. However, most current studies were conducted using CLA isomer mixtures. Only two studies performed on an animal model examined the differences between the cis-9, trans-11 and trans-10, cis-12 isomers, and found no direct effects on bones [48,49]. On the other hand, changes in body composition and anthropometric parameters may affect bone mass, as body weight reduction is associated with declining BMD [50]. However, Gaullier et al. [21] observed that after 12 months of intervention, CLA treatment significantly reduced body mass and body fat in comparison to the placebo group; however, no differences in bone mineral mass were detected. An essential research aspect of using a placebo is selecting an appropriate neutral substance that is inactive and does not affect the health of the patients. In our study, sunflower oil was used, as it shows similar organoleptic properties and energy values compared to CLA. Sunflower oil has also been used as a placebo in several trials [23,41,42]. In other studies, olive oil [19,20,21] or palm/bean oil were used [39].

It is worth noting some of the limitations of this study, including small sample size and a short intervention period. The bone remodelling cycle lasts a minimum of 100 days. Hence, our 90-day observation period may not have been sufficient to conclude how long-term supplementation may affect bones [51]. Moreover, we neither evaluated CLA levels in the blood nor estimated the effect of CLA supplementation on markers of bone formation and resorption. Additionally, we did not assess the subjects’ menopausal status to consider the hormonal influence on bone health. However, we speculate that the effect of CLA supplementation may differ in women depending on their menopausal status. Furthermore, information about tobacco, alcohol and medication use, family history and physical activity levels were not collected. Moreover, vitamin D and parathormone levels were not measured.

On the other hand, this was a well-designed double-blind randomised controlled trial that was performed according to CONSORT guidelines [30], in which we used strict inclusion and exclusion criteria allowing us to recruit a homogenous study population.

## 5. Conclusions

A three-month CLA supplementation in overweight and obese women did not affect bone health. However, the short intervention period limited our findings. Therefore, longer studies assessing the effect of CLA supplementation on densitometric parameters are needed.

## Figures and Tables

**Table 1:** Inclusion and exclusion criteria to the study.

Inclusion Criteria	Exclusion Criteria
Sex: women Age ≥ 18 years old BMI ≥ 25 kg/m^2^ Stable body weight during the last three months (±3 kg)	History of chronic diseases (e.g., celiac disease, type 2 diabetes mellitus, and hepatic and pancreatic) Pregnant and breastfeeding women Subjects who previously took CLA supplementation or dietary supplements which interfered with fat digestion or absorption

BMI—body mass index; CLA—conjugated linoleic acid.

**Table 2:** Content of fatty acids in capsules containing placebo and CLA.

	CLA [%]	Placebo [%]
Cis-9, trans-11 isomer	40	0
Trans-10, cis-12 isomer	40	0
Palmitic acid (C16:0)	3.6	4.8
Stearic acid (C18:0)	1.1	2.1
Oleic acid (C18:1)	12.9	10.2
Linoleic acid (C18:2)	1.2	61.2
α-linolenic acid (C18:3)	0.8	21.7
Arachidic acid (C20:0)	0.4	0

CLA—conjugated linoleic acid.

**Table 3:** Anthropometric characteristics of the study population.

	CLA Group (*n* = 37)		Placebo Group (*n* = 37)		*p*
	Mean ± SD	Median (IQR)	Mean ± SD	Median (IQR)	
Age [years]	51.8 ± 10.2	54 (43–59)	51.7 ± 11.4	54 (45–61)	0.9914
Weight [kg]	90.9 ± 12.2	90.0 (80.1–99.6)	93.3 ± 12.2	91.6 (85.4–101.0)	0.3546
Height [cm]	163 ± 4	163 (160–166)	163 ± 6	163 (159–167)	0.7067
BMI [kg/m^2^]	34.38 ± 4.28	34.00 (30.70–37.58)	34.99 ± 4.04	35.36 (31.75–38.62)	0.5049
Waist circumference [cm]	118.4 ± 10.7	116.0 (110.0–127.0)	119.6 ± 10.0	119.0 (111.5–125.5)	0.5983
Hip circumference [cm]	108.5 ± 7.0	108.0 (104.0–114.0)	109.1 ± 9.0	109.0 (103.0–115.0)	0.8298
Fat mass [%]	45.0 ± 4.3	44.9 (42.2–48.6)	43.8 ± 4.4	43.3 (41.3–46.9)	0.2489

BMI—body mass index; CLA—conjugated linoleic acid; IQR—interquartile range.

**Table 4:** Comparison of densitometric parameters between CLA and placebo groups before the intervention.

		Pre-Intervention					Post-Intervention					*p* ^1^	*p* ^2^
		CLA Group (*n* = 32)		Placebo Group (*n* = 30)		*p*	CLA Group (*n* = 32)		Placebo Group (*n* = 30)		*p*		
		Mean ± SD	Median (IQR)	Mean ± SD	Median (IQR)		Mean ± SD	Median (IQR)	Mean ± SD	Median (IQR)			
Total body	BMD [g/cm^2^]	1.16 ± 0.08	1.17 (1.11–1.22)	1.16 ± 0.11	1.15 (1.07–1.24)	0.9788	1.16 ± 0.07	1.17 (1.11–1.22)	1.17 ± 0.11	1.14 (1.09–1.22)	0.9161	0.7435	0.6733
	BMC [g]	2370.72 ± 247.84	2333.13 (2201.23–2534.94)	2428.63 ± 407.93	2365.75 (2168.07–2589.96)	0.8284	2376.10 ± 242.48	2340.67 (2242.62–2519.77)	2432.90 ± 415.99	2349.77 (2204.44–2627.32)	0.7743	0.9851	0.5577
Lumbar spine (L1–L4)	BMD [g/cm^2^]	1.03 ± 0.15	1.04 (0.94–1.08)	1.06 ± 0.20	1.04 (0.91–1.13)	0.7743	1.06 ± 0.15	1.06 (0.98–1.12)	1.03 ± 0.16	1.05 (0.93–1.12)	0.5533	0.0397	0.9018
	BMC [g]	58.82 ± 9.37	59.46 (52.87–63.06)	61.28 ± 13.59	58.89 (51.13–67.51)	0.5998	60.14 ± 9.71	60.10 (53.03–66.18)	61.45 ± 14.75	59.45 (53.41–67.55)	0.7530	0.0100	0.4284
Femoral neck	BMD [g/cm^2^]	0.87 ± 0.12	0.87 (0.82–0.92)	0.86 ± 0.14	0.85 (0.76–0.91)	0.7556	0.87 ± 0.11	0.87 (0.83–0.93)	0.87 ± 0.13	0.84 (0.78–0.93)	0.9891	0.4774	0.3223
	BMC [g]	4.22 ± 0.63	4.24 (3.87–4.56)	4.28 ± 0.83	4.07 (3.72–4.72)	0.7428	4.23 ± 0.58	4.40 (3.80–4.60)	4.23 ± 0.79	4.16 (3.84—4.71)	0.9822	0.8825	0.1722

BMC—bone mineral content; BMD—bone mineral density; CLA—conjugated linoleic acid; IQR—interquartile range; SD—standard deviation; ^1^ CLA group: pre- vs. post-intervention; ^2^ Placebo group: pre- vs. post-intervention.

**Table 5:** Comparison of differences in densitometric parameters between CLA and placebo groups.

		CLA Group (*n* = 32)		Placebo Group (*n* = 30)		*p*
		Mean ± SD	Median (IQR)	Mean ± SD	Median (IQR)	
Total body	Δ BMD [g/cm^2^]	0.00 ± 0.04	0.00 (−0.02–0.01)	0.00 ± 0.02	0.00 (−0.01–0.01)	0.5614
	Δ BMC [g]	5.37 ± 69.55	5.02 (−38.94–35.21)	4.27 ± 61.16	10.83 (−33.52–37.70)	0.6096
Lumbar spine (L1–L4)	Δ BMD [g/cm^2^]	0.03 ± 0.06	0.01 (−0.01–0.04)	−0.02 ± 0.13	−0.01 (−0.03–0.03)	0.1201
	Δ BMC [g]	1.32 ± 2.73	0.97 (−0.24–2.02)	0.18 ± 4.89	0.33 (−1.11–2.39)	0.3514
Femoral neck	Δ BMD [g/cm^2^]	0.00 ± 0.06	0.00 (−0.03–0.02)	0.01 ± 0.03	0.01 (−0.01–0.02)	0.2358
	Δ BMC [g]	0.01 ± 0.28	0.03 (−0.09–0.14)	−0.06 ± 0.23	0.00 (−0.19–0.07)	0.3225

BMC—bone mineral content; BMD—bone mineral density; CLA—conjugated linoleic acid; IQR—interquartile range; SD—standard deviation.

## Data Availability

The data presented in this study are available on request from the corresponding author (J.W.). The data cannot be publicly accessed as the result of disagreements among the study participants.

## Supplementary Materials

The following supporting information can be downloaded at: https://www.mdpi.com/article/10.3390/medicina59091690/s1, Table S1: CONSORT 2010 checklist of information to include when reporting a randomised trial.
